# Behavioural and psychological characteristics in Pitt-Hopkins syndrome: a comparison with Angelman and Cornelia de Lange syndromes

**DOI:** 10.1186/s11689-019-9282-0

**Published:** 2019-10-05

**Authors:** Alice Watkins, Stacey Bissell, Jo Moss, Chris Oliver, Jill Clayton-Smith, Lorraine Haye, Mary Heald, Alice Welham

**Affiliations:** 1 Cerebra Centre for Neurodevelopmental Disorders, School of Psychology, University of Birmingham, Birmingham, UK; 2 Great Ormond Street Institute of Child Health, University College London, London, UK; 3 Institute of Cognitive Neuroscience, University College London, London, UK; 4 Division of Evolution & Genomic Sciences, St Mary’s Hospital, Manchester, UK; 5 Department of Psychology, University of Leicester, Leicester, UK

**Keywords:** Angelman syndrome, Autism spectrum disorder, Behavioural phenotype, Cornelia de Lange syndrome, Pitt-Hopkins syndrome

## Abstract

**Background:**

Pitt-Hopkins syndrome (PTHS) is a genetic neurodevelopmental disorder associated with intellectual disability. Although the genetic mechanisms underlying the disorder have been identified, description of its behavioural phenotype is in its infancy. In this study, reported behavioural and psychological characteristics of individuals with PTHS were investigated in comparison with the reported behaviour of age-matched individuals with Angelman syndrome (AS) and Cornelia de Lange syndrome (CdLS).

**Methods:**

Questionnaire data were collected from parents/caregivers of individuals with PTHS (*n* = 24), assessing behaviours associated with autism spectrum disorder (ASD), sociability, mood, repetitive behaviour, sensory processing, challenging behaviours and overactivity and impulsivity. For most measures, data were compared to data for people with AS (*n* = 24) and CdLS (*n* = 24) individually matched by adaptive ability, age and sex.

**Results:**

Individuals with PTHS evidenced significantly higher levels of difficulties with social communication and reciprocal social interaction than individuals with AS, with 21 of 22 participants with PTHS meeting criteria indicative of ASD on a screening instrument. Individuals with PTHS were reported to be less sociable with familiar and unfamiliar people than individuals with AS, but more sociable with unfamiliar people than individuals with CdLS. Data also suggested areas of atypicality in sensory experiences. Challenging behaviours were reported frequently in PTHS, with self-injury (70.8%) occurring at significantly higher rates than in AS (41.7%) and aggression (54.2%) occurring at significantly higher rates than in CdLS (25%). Individuals with PTHS also evidenced lower reported mood than individuals with AS.

**Conclusions:**

Behaviours which may be characteristic of PTHS include those associated with ASD, including deficits in social communication and reciprocal social interaction. High rates of aggression and self-injurious behaviour compared to other genetic syndrome groups are of potential clinical significance and warrant further investigation. An atypical sensory profile may also be evident in PTHS. The specific aetiology of and relationships between different behavioural and psychological atypicalities in PTHS, and effective clinical management of these, present potential topics for future research.

## Background

Pitt-Hopkins syndrome (PTHS, OMIM #610954) is a genetic neurodevelopmental disorder associated with an abnormal expression of the basic helix-loop-helix transcription factor 4 gene (TCF4) located on chromosome 18q21 (OMIM #602272). PTHS was first identified in 1978 by Pitt and Hopkins [1], who described the syndrome in two unrelated patients presenting with a characteristic facial gestalt (comprising a squared forehead, deep-set eyes, a wide mouth, tented upper lip, full lower lip and a broad and/or beaked nasal bridge), developmental delay and abnormal breathing patterns [2–4]. Although the definitive prevalence of PTHS is unknown, prevalence estimates lie between 1 in 225,000 and 1 in 300,000 based on the known number of affected individuals [5].

PTHS is commonly associated with intellectual disability (ID), with severe ID identified in 100% of a cohort of 101 patients with PTHS [6]. Impaired language development is also reported frequently, with many individuals presenting with absent language or speech limited to a few words [4, 7]. Hypotonia and delays in motor development are also prevalent, most notably delayed or absent walking, impaired motor coordination and ataxia [6, 8].

Recent years have seen progress towards characterising the specific behavioural characteristics associated with PTHS, based on a number of case and cohort studies (e.g. [4, 6, 9, 10], see [7] for a review). The first international consensus statement about PTHS [5] draws together current understanding of these with directions for the diagnosis and management of PTHS. The temperament of individuals with PTHS is often described as amiable and happy, characterised by an “easy-going” demeanour (87%) [5, 7] and smiling appearance (89%) [4, 6]. Indeed, it has been noted that there are similarities with Angelman syndrome (AS) [4, 5], a genetic condition in which “excessive” smiling has been established as a phenotypic behavioural feature [7, 11, 12]. Furthermore, pathogenic variants of the TCF4 gene have previously been identified in cases of diagnosed AS (2%) [13], sometimes leading to delays in diagnosis [4]. However, systematic study of social characteristics using established measures with known psychometric properties have been rare (although see [6, 14]), and formal comparisons with AS are yet to be drawn. Alongside reports of specific social characteristics, a number of behavioural traits associated with autism spectrum disorder (ASD) are reportedly associated with PTHS. ASD is a complex diagnostic category, defined by repetitive behaviours/restricted interests and impairments in social affect, which itself is defined as difficulties with communication (verbal and non-verbal) and social interaction (e.g. [15, 16]). Consistent with possible ASD-related symptomatology, individuals with PTHS are reported to show impaired social interaction and communication, stereotyped movements, repetitive behaviours and difficulty with changes to daily routine [2, 5, 8, 14]. Whalen et al. [4] reported an overall repetitive behaviour prevalence of 94%. Reported stereotypies include: repetitive hand and finger movements (48% [4], 86% [3]), arm flapping (80%) [4], hand washing (45%) [4] and body rocking [14]. ASD occurs with elevated frequency in a number of genetic syndrome groups, including AS (34% [17]) and Cornelia de Lange syndrome (CdLS, 43% [17]), and it has been noted that having a genetic syndrome itself increases the probability with which a person will meet diagnostic criteria for ASD [17]. In addition, the qualitative nature of ASD presentation may vary between genetic syndrome groups (e.g. [18, 19]). The degree to which ASD-related behaviours present in excess of what may be expected for developmental level has been debated for a number of syndrome groups (see [20]). In the case of PTHS, comparison with matched-ability groups with other genetic syndromes may help elucidate the possible presence and nature of ASD-related behaviours.

In addition to the social and repetitive elements of ASD, sensory processing differences have been increasingly foregrounded in ASD research (e.g. [21–23]), with the DSM 5 including sensory processing factors in the diagnostic criteria for ASD [16]. To date, the sensory processing profile of PTHS is yet to be explicitly researched in individuals with PTHS, and the need for further study of this topic has been highlighted within the international consensus statement [5].

Further areas of potential behavioural atypicality and difficulty for people with PTHS include high rates of anxiety (81%) [4], aggression (40–50%) [2, 7, 14] and self-injurious behaviours (e.g. pinching, hand biting, hitting oneself) [2, 6] and some reports of attention deficit hyperactivity disorder (ADHD) [7]. Each of these behaviours has also been associated to varying degrees with other genetic neurodevelopmental syndromes. For example, over 70% of people with CdLS are reported to display self-injury [24], and a number of studies have also found high levels of overactivity in this syndrome group (e.g. [25–27]).

The set of behaviours associated with a specific genetic syndrome has often been referred to as a “behavioural phenotype” [28, 29], defined as the behaviours which occur more often in individuals with a specific syndrome than in people without this syndrome [29, 30]. Characterisation of behavioural phenotypes is aided by the use of standardised measures suitable for people with ID (e.g. [31–33]). In addition, comparisons with suitable groups, including those with other genetic syndromes also associated with ID, has been foregrounded as crucial in the study of behavioural phenotypes [34]. To date, much of the PTHS behavioural research is in the form of case study or case series methodology (e.g. [10], see [7] for a review). Where larger cohort studies have been conducted (e.g. [4, 6, 9], see [7] for a review), a lack of cross-syndrome comparisons with other genetic syndrome groups limits interpretation.

### Current study

The current study assessed reported behavioural characteristics in a cohort of people diagnosed with PTHS, using measures with established psychometric properties and history of utility for investigation of behavioural phenotypes of genetic neurodevelopmental syndromes associated with ID (e.g. [18, 19, 24, 31, 35]). Measures were included of ASD-related characteristics, sociability, mood, repetitive behaviours, sensory processing, challenging behaviours, overactivity and impulsivity.

Comparisons were made with age- and ability-matched participants from two other genetic syndrome groups: AS and CdLS. Selection of these two groups for comparison was based on a number of factors. First, both AS and CdLS are associated with ID which may be profound [12, 25], potentially increasing the likelihood of highlighting behavioural features of PTHS which are not solely related to ID or to having a genetic syndrome per se. In addition, the behavioural phenotypes of AS and CdLS are both relatively well established (e.g. [12, 25, 36–41]), allowing appropriate contextualisation of data for PTHS. These phenotypes are summarised, alongside genetic information and information about physical characteristics, in Table 1. AS and CdLS are also divergent on some behavioural aspects, thus providing significant points of contrast. For example, both CdLS and AS are frequently associated with ASD characteristics [17], but the specific profiles of ASD-related behaviours differ. For instance, in relation to behaviour in social contexts, positive affect during social interactions is often reported in AS alongside impairments in social affect contributing to meeting diagnostic criteria for ASD [47]. In CdLS, impaired communication and social interaction skills are prominent [19], with difficulties putatively related at least in part to high levels of social anxiety [48]. As in CdLS, individuals with PTHS are reported to show impaired social interaction [5, 6, 14], although systematic study of this is lacking. However, the “happy and amiable” demeanour frequently reported in PTHS appears to overlap with the phenotypic smiling and laughter reported of AS [7, 11, 12]. CdLS and AS also differ notably in associated mood, poor mood and high anxiety [25, 49] (social anxiety in particular) [50, 51] associated with CdLS and elevated mood associated with AS [12]. Both AS and CdLS are associated with challenging behaviour, but AS is associated with higher rates of aggression compared to CdLS (73% > 40.2%), and CdLS is associated with higher rates of self-injury than AS (70.3% > 45.1%) [24].

**Table 1:** The genetic mechanism, estimated prevalence and suggested behavioural phenotypes of the comparison syndrome groups AS and CdLS

Syndrome	Genetic mechanisms	Estimated prevalence	Behavioural phenotype
Pitt-Hopkins syndrome (PTHS)	Deletion of or variants in the TCF4 gene located at 18q21.2 that encodes transcription factor 4 [5].	Estimated as 1 in 225,000 to 300,000 [5].	ID, speech and language impairment, anxiety, self-injurious behaviour, aggression, repetitive behaviour and ASD [4–7].
Angelman syndrome (AS)	Deficiency or disruption to the UBE3A gene located on chromosome 15. Approximately 70% of AS cases are caused by de novo maternal deletions at 15q11-q13 [42].	Estimated at 1 in 20,000 in the population [43].	Severe ID, speech, and language impairment, ataxic movement/movement or balance disorder (e.g. hypermotoric behaviour), enhanced laughter/smiling, happy demeanour, short attention spans and aggression [12].
Cornelia de Lange syndrome (CdLS)	Heterozygous mutation of the NIPBL gene, located on chromosome 5p13 (approximately 65% of cases, with further cases being due to mosaicism). Less commonly caused by mutations in SMC3, SMC1A, HDAC8 and RED21 genes (11% of cases) [44].	Estimated between 1 in 10,000 and 1 in 30,000 live births [45].	ID, speech and language delay, self-injurious behaviour, autistic features, repetitive behaviours, aggression and hyperactivity [25, 36, 40, 41, 46].

## Methods

### Recruitment

Participants with PTHS were recruited via the Pitt Hopkins UK family support group. All families who had provided consent to be contacted by the syndrome support group were invited to take part in the present study via research advertisements shared by email and social media platforms. Twenty-four families responded to this invitation and provided consent to participate in the present study.

### Procedure

Parents/caregivers of individuals with PTHS were invited to complete online questionnaires, using LimeSurvey 2.00+ software. The online survey contained information sheets, consent forms, a background information questionnaire including demographic questions and questions about the person’s diagnosis and genetic mutation and informant-report questionnaires assessing behavioural, psychological, social and physical characteristics commonly reported in ID populations.

### Participants

All participants had a diagnosis of PTHS, made by a clinical geneticist, paediatrician, or via medical research participation. Reported genetic mutations were 11 unspecified mutations of TCF4, nine deletions, one frameshift mutation, one translocation, one sequence repetition and one splice site mutation, ascertained through parent-report of syndrome diagnosis details (including the genetic mechanism if known, date of diagnosis and diagnostic information).

The mean age of the PTHS group was 11.2 years (SD = 7.8 years, range = 1–30 years) and 50% of the sample were male (*n* = 12). Eleven participants (45.8%) were fully mobile (defined as able to walk unaided), and two participants (8.3%) were verbal (defined as able to speak or sign more than 30 words). Nine participants (37.5%) had normal vision, 14 (58.3%) had poor vision and one participant (4.2%) was blind/almost blind. Twenty-three participants (95.8%) had normal hearing and one participant (4.2%) was deaf/almost deaf.

The comparison groups had previously participated in research projects at the Cerebra Centre for Neurodevelopmental Disorders, University of Birmingham, and had provided consent for their data to be utilised in future research studies. Individuals with CdLS and AS were matched to individuals with PTHS first by level of ability, according to the self-help subscale of the Wessex scale [52] (self-help score +/− 1 point), then by age (+/− 3 years), verbal ability (verbal or non-verbal), mobility and sex. Every participant with PTHS but one was matched with equal scores for ability to individuals with AS and CdLS [52]. The age of participants was then prioritised before matching for verbal ability, mobility and sex.

### Measures

The background information questionnaire provided details relating to sex, age, verbal ability, mobility and any health problems encountered within the last 6 months. Additional parent-report questionnaires included the Wessex Questionnaire (WQ) [52], Social Communication Questionnaire Lifetime Version (SCQ) [53], the Sociability Questionnaire for people with Intellectual Disability (SQID) [31], the Challenging Behaviour Questionnaire (CBQ) [36], the Mood, Interest and Pleasure Questionnaire-Short Form (MIPQ) [32], the Repetitive Behaviour Questionnaire (RBQ) [33], the Activity Questionnaire (TAQ) [54], Health Questionnaire (HQ) [55], and the Sensory Experience Questionnaire (SEQ) [56]. See Table 2 for descriptions and psychometric properties of assessments used.

**Table 2:** Description and psychometric properties of assessments used

Assessment [authors]	Description of assessment	Scoring	Psychometric properties
Wessex Questionnaire (WQ) [52]	The questionnaire assesses ability in individuals with ID. The following subscales are assessed: continence, mobility, self-help skills, speech and literacy. Within the current study, “self-help” was used as a proxy measure to assess the degree of ability.	On the self-help subscale, individuals are rated on their ability to feed, wash and dress themselves. For each task, they are scored on a 3-point scale. The total self-help score ranges from 3 to 9: total score of 3–5 (“not able”), 6–7 (“partly able”) and 8–9 (“able”).	The scale has modest inter-rater reliability at subscale level for both children and adults ranging from 78% (self-help, literacy) to 92% (mobility) [52].
Social Communication Questionnaire Lifetime Version (SCQ) [57]	The parent-report questionnaire is used to measure ASD symptomatology and is a screening tool for ASD based on the Autism Diagnostic Interview. It consists of 40 items grouped into three subscales: communication, social interaction and repetitive and stereotyped behaviours. The lifetime version is completed in regards to an individual’s full developmental history.	The 40 items all require a yes/no response. Total scores range from 0 to 39. A cut-off score of > 15 indicates possible ASD and > 22 indicates possible autism.	The cut-off point of > 15 was found to distinguish pervasive developmental disorder (PDD) individuals from other diagnoses with a specificity of .80 and a sensitivity of .96 (excluding individuals with ID) and distinguished individuals with autism from people with ID with a specificity of .67 and sensitivity of .96. The higher cut-off of > 22 was necessary to distinguish between autism individuals with PDD with a specificity of .60 and a sensitivity of .75. The scale has good concurrent validity with the Autism Diagnostic Interview and with the Autism Diagnostic Observation Schedule [57, 58]. Internal consistency is good [19, 57].
Sociability Questionnaire for people with Intellectual Disability (SQID) [31]	The informant-based questionnaire assesses the sociability in children and adults and is appropriate for individuals with ID. The questionnaire contains 25 items including 13 categories and is to be completed regarding an individual’s typical behaviour across social situations with familiar and unfamiliar people and considers the possible indication of selective mutism.	The SQID consists of 25 items, 21 are on a 7-point scale (e.g. items 1–17: range from “very shy” to “very sociable”) and four are yes/no responses. Total scores evaluate the effect of social context on an individual’s sociability with a familiar or unfamiliar person. A higher score indicates more sociability.	The scale has a satisfactory inter-rater reliability for item level between .43 and .80 (Spearman’s coefficient > .60) for Q1-21. Kappa values for categorical items were .96, .44 and .51 (Q22, 24, 25). The scale also has good concurrent validity with the Child Sociability Rating Scale (CSRS [19]) (rs = .36 to .52; all *p* = .01).
Challenging Behaviour Questionnaire (CBQ) [36]	Part one of the questionnaire assesses the presence of self-injury, physical aggression, verbal aggression, destruction of property and stereotyped behaviour over the course of the previous month. Part two assesses the severity of each type of challenging behaviour.	Part one is based on a yes/no basis. Part two requires responses on a 4–5-point scale (14 items). Item scores are summed to provide an overall severity score. Lower scores indicate less severe behaviour.	The scale has good inter-rater reliability (range of *α* = .62 to .72) [36]. Concurrent validity of the total scores of the CBQ and the Aberrant Behavior Checklist (ABC [59]) was good (0.56, *p* < .01) [60].
The Mood, Interest and Pleasure Questionnaire-Short Form (MIPQ) [32]	The informant based questionnaire is appropriate for use for individuals with ID and assesses two main subscales of mood and interest and pleasure.	The short form consists of 12 items (6 items for each subscale). Items are rated on a 5-point scale, and total scores range between 0 and 48. A higher score indicates positive affect and higher levels of interest and pleasure.	The short form version of the MIPQ has good internal consistency (*α* total = .88; *α* Mood = .79; *α* Interest and Pleasure = .87), test-retest (.97) and inter-rater reliability (.85). Concurrent validity between the MIPQ and the ABC [59] ranged from medium to strong (0.36–0.73; *p* < .001).
Repetitive Behaviour Questionnaire (RBQ) [33]	The informant-based questionnaire is appropriate for use for children and adults with ID and examines the frequency of repetitive behaviours over the last month. The scale consists of 19 items including five subscales: stereotyped behaviour, compulsive behaviour, insistence on sameness, restricted preferences and repetitive speech.	Informants rate the frequency of each behaviour over the last month. Scores are rated on a 5-point scale from “never” (0) to “more than once a day” (5). A verbal score ranges between 0 and 76 and a nonverbal score ranges between 0 and 60 (4 items are only applicable to verbal individuals). Behaviours occurring “once a day” or “more than once a day” are deemed to be of clinical importance. A clinical cut-off is obtained if an individual has a score of three or more on at least one item within a subscale.	Item-level inter-rater and test-retest reliability and validity are good Spearman’s coefficients for inter-rater reliability range from .46 to .80 at item-level (73% > .60). Spearman’s coefficients for test-retest reliability range from .61 to .93 at item level (52.6% > .80). The scale has good concurrent validity and content validity between the RBQ and the repetitive subscale of the Autism Screening Questionnaire (ASQ [57]) (.6; *p* < .001). There is good Internal consistency at full-scale level (*α* > .80) and for the stereotyped behaviour and compulsive behaviour subscales (*α* > .70), this was lower for restricted preferences, repetitive speech and insistence on sameness subscales (*α* = .50, .54 and .65) [61].
The Activity Questionnaire (TAQ) [54]	The information-based questionnaire assesses behaviours that indicate overactivity and impulsivity and is suitable for use for people with ID. The questionnaire consists of 18 items grouped into three subscales: over-activity, impulsive speech and impulsivity.	The score range for over-activity is 0–36, impulsive speech 0–24 and impulsivity 0–24. Impulsive speech is not calculated for individuals who are non-verbal. Items are scored on a 5-point scale of “never/almost never” (0) to “always/almost all of the time” (5) to assess activity frequency. Scores of 32 for overactivity and 24 for impulsivity are identified as “abnormally high” (at or above the 95th percentile).	The scale has a good item level inter-rater reliability (mean of .56, range = .31 to.75) and test-retest reliability (mean of .75, range = .60 to .90). Inter-rater and test-retest reliability for subscales and total score were above .70 [54].
Sensory Experience Questionnaire- Short form, Version 2.1 (SEQ) [56]	The questionnaire examines the frequency of sensory behaviours across sensory response patterns (Hypo-Social, Hypo-Nonsocial, Hyper-Social and Hyper-Nonsocial), five sensory modalities (Tactile, Auditory, Visual, Gustatory, and Vestibular) and across social or non-social contexts.	The scale consists of 41 items (counting sub-items) rated on a 5-point scale of “almost never” (1) to “almost always” (5). A total score is obtained and sub-scores for sensory patterns (Hypo-responsiveness, Hyper-Responsiveness and Sensory Seeking), a score for each modality and for social and non-social contexts. A higher score is indicative of more severe sensory symptoms.	The overall internal consistency is *α* = .80 [62] and for subscales Hyper-Responsiveness *α* = .73, Hypo-Responsiveness *α* = .75, Sensory Seeking *α* = .80, Social *α* = .69, Non-Social *α* = .78. The test-retest reliability is .92 (intraclass correlation coefficient) [63]. Concurrent validity between the SEQ and the Sensory Processing Assessment (SPA) indicated significant correlations between Hypo-Responsiveness subscales and Hyper-Responsiveness scales [64].
Health Questionnaire (HQ) [55]	The questionnaire looks at the presence and subsequently the severity of 15 different health difficulties, with subsections assessing health difficulties over the entire lifetime and over the course of the last month.	The scale consists of 32 items. Severity of each reported health difficulty is rated on a 3-point scale of never (0) to severe (3). An overall health score is determined by summing the total for both time periods. A higher score indicates greater severity.	Inter-rater reliability for health difficulties reported across the individuals lifetime was *α* = 0.72 and for those present within the last month were *α* = 0.76. Internal consistency is found to be good (*α* = 0.77) for the overall health score [55].

#### Data analysis

Due to violations of assumptions of parametric tests (including of normality and homogeneity of variance), non-parametric tests were used throughout.

### Matched group comparisons

In order to compare individuals with PTHS with matched AS and CdLS groups, a series of Kruskal-Wallis tests were conducted to analyse group differences in total and subscale scores of questionnaires. Participants under the age of 4 years were excluded from SCQ data analysis, as this measure is not validated in children younger than 4 years [53]. Post hoc Mann-Whitney *U* analyses were conducted to identify significant differences between PTHS and matched syndrome groups individually. Categorical data derived from the SCQ (meeting cut-off scores for ASD; SCQ scores > 15) and CBQ (yes/no presentation of behaviours within the previous month) were compared between groups using chi-square analyses and Fisher’s exact tests. Effect sizes were calculated in concordance with guidelines for non-parametric tests [65] and interpreted according to guidelines by Cohen [66].

### Sociability and sensory experiences

Data for the SQID and the SEQ were not available for matched comparison groups. The analysis was therefore conducted in relation to previously published data. To assess sociability, single-sample Wilcoxon tests were performed to compare PTHS median scores for totals and subscales from the SQID with median scores presented by Moss and colleagues [31] for individuals with AS and CdLS. To explore sensory experiences in individuals with PTHS, SEQ median and interquartile ranges were reported and categorised into “typical”, “at risk” and “deficient” range according to Baranek’s [56] criterion cut-off points based upon data for typically developing participants.^a^

### Alpha

The relatively large number of group comparisons used in this study increases the likelihood of type 1 errors (inappropriately rejecting the null hypothesis). However, low power due to the relatively low n increases the likelihood of type 2 errors (inappropriately accepting the null hypothesis), which may involve overlooking clinically important group differences. Therefore, effects at *p* < 0.05 were reported as significant, but interpretation should be cautious, with attention also paid to effect sizes.

## Results

### Cross-syndrome comparison: demographic characteristics

There were no significant differences between groups in adaptive ability, age, verbal ability, mobility or sex (see Table 3), indicating individuals with PTHS were well matched to both AS and CdLS groups. The average score on the self-help subscale of the Wessex [52] was low (mean 4; total scores range from 3 to 9, see Table 3), estimating limited self-help abilities. Participants with PTHS, AS and CdLS showed similar low ability across skill areas including washing, dressing and feeding ability. There were fewer individuals in the PTHS group with normal vision than in the AS group (*χ*^2^(1) = 12.16, *p* < .001) and more individuals with PTHS with normal hearing compared to the CdLS group (*χ*^2^(1) = 19.5, *p* < .001).

**Table 3:** Demographic characteristics and statistical analyses for participant groups: PTHS and matched AS and CdLS

Demographic characteristic		PTHS, *n* = 24	AS, *n* = 24	CdLS, *n* = 24	Group comparison: chi-squared and Mann-Whitney *U*		
					Comparison	*χ*^2^/*U*	*p* value
Self-help score^a^	Mean (SD)	4.04 (.62)	4.04 (.62)	4 (.51)	PTHS ≈ AS	288	1.00
	Median (IQR)	4 (0)	4 (0)	4 (0)	PTHS ≈ CdLS	287	.967
	Range	3–6	3–6	3–5			
Age	Mean (SD)	11.2 (7.8)	10.9 (7.3)	11.3 (7.7)	PTHS ≈ AS	282	.901
	Median (IQR)	8.5 (11)	8.5 (11)	9 (11)	PTHS ≈ CdLS	285	.951
	Range	1–30	2–27	1–30			
Verbal (able to speak more than 30 signs/words) (%)		2 (8.3)	0 (0)	1 (4.2)	PTHS ≈ AS	2.09	.490^b^
					PTHS ≈ CdLS	.356	1.00^b^
Mobile (able to walk unaided) (%)		11 (45.8)	11 (45.8)	16 (66.7)	PTHS ≈ AS	.000	1.00
					PTHS ≈ CdLS	2.12	.146
% male		50	47.8	41.7	PTHS ≈ AS	.020	.880
					PTHS ≈ CdLS	.336	.560
Vision^a^ (categorised as “normal vision”)		9 (37.5)	20 (87)	14 (60.9)	PTHS < AS	12.16	< .001
					PTHS ≈ CdLS	2.57	.109
Hearing^a^ (categorised as “normal hearing”)		23 (95.8)	24 (100)	8 (34.8)	PTHS ≈ AS	1.02	1.00^b^
					PTHS > CdLS	19.50	< .001

*IQR* interquartile range

^a^Derived from the Wessex Scale [52]

^b^Fishers exact *p* value reported as 50% had an expected count < 5

### Physical health

The most frequently reported lifetime health difficulties were gastrointestinal problems (*n* = 20, 83.3%), described as moderate severity by 29.2%, with seven individuals (35%) needing corrective treatment. Other frequent health difficulties were epilepsy/seizures (*n* = 12, 50%), ear problems (*n* = 12, 50%) and skin problems (*n* = 11, 45.8%). See Table 4 for details regarding health difficulties experienced by individuals with PTHS.

**Table 4:** Health difficulties experienced by individuals with Pitt-Hopkins syndrome as taken from the Health Questionnaire

		Never/no	Mild	Moderate	Severe	Corrective surgery/treatment/medication?
		*N* (%)	*N* (%)	*N* (%)	*N* (%)	*N* (%)
Eye problems	Lifetime	17 (70.8)	2 (8.3)	3 (12.5)	2 (8.3)	5 (71.4)
	Last month	23 (95.8)	1 (4.2)	–	–	
Ear problems	Lifetime	12 (50)	8 (33.3)	3 (12.5)	1 (4.2)	4 (33.3)
	Last month	23 (95.8)	–	1 (4.2)	–	
Dental problems	Lifetime	20 (83.3)	1 (4.2)	2 (8.3)	1 (4.2)	1 (25)
	Last month	23 (95.8)	1 (4.2)	–	–	
Cleft palate	Lifetime	24 (100)	–	–	–	–
	Last month	23 (95.8)	1 (4.2)	–	–	
Gastrointestinal problems	Lifetime	4 (16.7)	9 (37.5)	7 (29.2)	4 (16.7)	7 (35)
	Last month	11 (45.8)	9 (37.5)	3 (12.5)	1 (4.2)	
Bowel problems	Lifetime	5 (20.8)	14 (58.3)	2 (8.3)	3 (12.5)	3 (15.8)
	Last month	13 (54.2)	7 (29.2)	3 (12.5)	1 (4.2)	
Heart abnormalities or circulatory problems	Lifetime	22 (91.7)	1 (4.2)	1 (4.2)	–	–
	Last month	24 (100)	–	–	–	
Problems with genitalia	Lifetime	21 (87.5)	1 (4.2)	2 (8.3)	–	2 (66.7)
	Last month	24 (100)	–	–	–	
Hernia	Lifetime	23 (95.8)	–	–	1 (4.2)	1 (100)
	Last month	23 (95.8)	1 (4.2)	–	–	
Limb abnormalities	Lifetime	24 (100)	–	–	–	–
	Last month	24 (100)	–	–	–	
Epilepsy/seizures	Lifetime	12 (50)	8 (33.3)	4 (16.7)	–	5 (41.7)
	Last month	20 (83.3)	2 (8.3)	2 (8.3)	–	
Lung or respiratory problems	Lifetime	20 (83.3)	3 (12.5)	1 (4.2)	–	3 (75)
	Last month	24 (100)	–	–	–	
Liver or kidney problems	Lifetime	24 (100)	–	–	–	–
	Last month	24 (100)	–	–	–	
Diabetes or thyroid function problems	Lifetime	24 (100)	–	–	–	–
	Last month	24 (100)	–	–	–	
Skin problems	Lifetime	13 (54.2)	7 (29.2)	3 (12.5)	1 (4.2)	5 (45.5)
	Last month	16 (66.7)	4 (16.7)	4 (16.7)	–	

### Autism spectrum disorder

There were significant between-group differences on the total score, *Communication* subscale and *Reciprocal Social Interaction* subscale of the SCQ (see Table 5). Individuals with PTHS showed significantly higher scores than those with AS on the total SCQ (*U* = 73.5, *p* < .001, *r* = .59, large effect size), the *Communication* subscale (*U* = 55, *p* < .001, *r* = .69, large effect size) and the *Reciprocal Social Interaction* subscale (*U* = 132, *p* = .009, *r* = .39, medium effect size). No significant differences were found between individuals with PTHS and CdLS on the total SCQ or any of the subscales. According to the SCQ, a significantly greater proportion of individuals with PTHS (95.5%) met the cut-off score to indicate possible ASD compared to AS (68.2%) (*χ*^2^ = 5.5 (1), *p* = .023), with a relative risk of 1.4 (95% CI, 1.04–1.89).

**Table 5:** Median and interquartile ranges of ASD-related behaviours and percentage meeting criteria for ASD from the SCQ for participant groups: PTHS, AS and CdLS

		PTHS	AS	CdLS	Kruskal-Wallis tests/chi-squared test			Post hoc comparison: Mann-Whitney *U* test/chi-squared test					
					h/*χ*^2^	df	*p* value	Comparison	*U*/*χ*^2^	df	*Z*	*p* value	Effect size *r*/relative risk (95% CI)
SCQ—total	*N*	22	22	18									
	Median	24	18	25	18.41	2	< .001	PTHS > AS	73.50	1	3.965	< .001	0.59 (large)
	IQR	3.75	7	7.57									
SCQ—communication	*N*	22	22	18									
	Median	8	6	6.93	25.70	2	< .001	PTHS > AS	55.00	1	4.555	< .001	0.69 (large)
	IQR	0.25	3	1.25									
SCQ—restricted, repetitive and stereotyped behaviours	*N*	22	22	22									
	Median	4	4	5	3.56	2	.169	N/A					
	IQR	3	2.25	2.63									
SCQ—reciprocal social interaction	*N*	22	22	18									
	Median	10.5	7	12	11.15	2	.004	PTHS > AS	132.00	1	2.598	.009	.39 (medium)
	IQR	4.25	5.25	5.25									
Met ASD cut-off	*N*	22	22	18									
	*N* (%)	21 (95.5)	15 (68.2)	16 (88.9)	5.85	2	.049^a^	PTHS > AS	5.50	1	–	.023	1.40 (1.04 to 1.89)^b^

^a^Fishers exact *p* value reported as 50% had an expected count < 5

^b^Relative risk of meeting criteria for ASD in AS relative to PTHS

### Sociability

Significant differences in sociability scores were evident, when data from the PTHS group were compared to data from AS and CdLS groups derived from Moss et al. [31], using the SQID (see Table 6). Individuals with PTHS displayed significantly lower sociability than those with AS on both *Unfamiliar* (*Z* = 2.88, *p* = .004) and *Familiar* (*Z* = 3.95, *p* < .001) total scores. However, individuals with PTHS had higher sociability scores than those with CdLS on *Unfamiliar* total scores (*Z* = 2.92, *p* = .004) suggesting individuals with PTHS show higher levels of sociability with unfamiliar adults relative to individuals with CdLS.

**Table 6:** Median and interquartile range for PTHS and comparison syndrome groups: AS and CdLS, derived from [31] and one-sample Wilcoxon test

Median scores		PTHS (*n* = 24)	AS (*n* = 66) [31]	CdLS (*n* = 98) [31]	One-sample Wilcoxon test				
					Comparison group [31]	*Z*	*p* value	PTHS significance	Effect size *r*
SQID familiar—total	Median (IQR)	42.5 (15.25)	53 (48–55)	41.5 (35–48)	AS	3.95	< .001	PTHS < AS	.57 (large)
					CdLS		.360		
SQID familiar—receive interaction	Median (IQR)	11.5 (3)	13 (12–14)	10 (8.75–12)	AS	3.21	.001	PTHS < AS	.46 (medium)
					CdLS	2.98	.003	PTHS > CdLS	.43 (medium)
SQID familiar—interaction	Median (IQR)	12 (4)	13.5 (12–14)	11 (10–13)	AS	2.90	.004	PTHS < AS	.42 (medium)
					CdLS		.072		
SQID familiar—approach or initiate interaction	Median (IQR)	8 (5)	13 (10–14)	10 (7–12)	AS	4.21	< .001	PTHS < AS	.61 (large)
					CdLS	2.47	.014	PTHS < CdLS	.36 (medium)
SQID familiar—performance	Median (IQR)	12 (3)	14 (12–14)	11 (9–13)	AS	3.83	< .001	PTHS < AS	.55 (large)
					CdLS		.528		
SQID unfamiliar—total	Median (IQR)	35.5 (12.75)	41 (31–48)	26 (13.5–35)	AS	2.88	.004	PTHS < AS	.42 (medium)
					CdLS	2.92	.004	PTHS > CdLS	.42 (medium)
SQID unfamiliar—receive interaction	Median (IQR)	9 (3)	10 (8–12)	6.5 (3–8)	AS		.108		
					CdLS	3.30	.001	PTHS > CdLS	.48 (medium)
SQID unfamiliar—interaction	Median (IQR)	9.5 (3)	11 (9–12)	7 (3–9)	AS	3.03	.002	PTHS < AS	.44 (medium)
					CdLS	2.95	.003	PTHS > CdLS	.43 (medium)
SQID unfamiliar—approach or initiate interaction	Median (IQR)	6 (4.75)	10 (6.75–12)	5 (4–7.5)	AS	3.72	< .001	PTHS < AS	.54 (large)
					CdLS	2.33	.020	PTHS > CdLS	.34 (medium)
SQID unfamiliar—performance	Median (IQR)	10 (3)	10 (7–13)	7 (2.75–9)	AS		.203		
					CdLS	2.16	.031	PTHS > CdLS	.31 (medium)

### Repetitive behaviour

There were no significant differences between PTHS, AS and CdLS groups on the RBQ total scores or any subscale score (*Stereotyped Behaviour*, *Compulsive Behaviour* or *Insistence on Sameness*, see Table 7). Items on the RBQ most frequently endorsed by individuals with PTHS were in the Stereotyped Behaviour domain, including object stereotypy (62.5%, all more than once a day), body stereotypy (54.2%, all more than once a day) and hand stereotypy (79.2%, all more than once a day). Overall, 75% of individuals with PTHS evidenced stereotyped behaviour within the previous month according to the CBQ (see Table 9).

**Table 7:** Median and interquartile ranges of behavioural characteristics, Kruskal-Wallis tests and post hoc Mann-Whitney *U* test analyses for participant groups: PTHS, AS and CdLS

		PTHS	AS	CdLS	Kruskal-Wallis tests			Post hoc comparison: Mann-Whitney *U* test					
					*h*	df	*p* value	Comparison	*U*	df	*Z*	*p* value	Effect size *r*
CBQ—Severity	*N*	17	10	19									
	Median	8	7	7	1.84	2	.4	N/A					
	IQR	3	3.75	3									
MIPQ—Mood	*N*	24	24	24									
	Median	19	22	18	24.89	2	< .001	PTHS < AS	105	1	3.819	< .001	.55 (large)
	IQR	3.75	1	4.75									
MIPQ—Interest and Pleasure	*N*	24	24	24									
	Median	17	17.5	15	2.07	2	.356	N/A					
	IQR	4.75	5.5	4.75									
MIPQ—Total	*N*	24	24	24									
	Median	36	39.5	34	9.87	2	.007	PTHS < AS	185.5	1	2.121	.034	.31 (medium)
	IQR	3.75	7	8.75									
RBQ—Total	*N*	22	23	22									
	Median	12	12	16.5	4.34	2	.114	N/A					
	IQR	6.5	10	24									
RBQ—Stereotyped Behaviour	*N*	24	23	24									
	Median	10.5	8	9.5	2.85	2	.241	N/A					
	IQR	4.5	8	6									
RBQ—Compulsive Behaviour	*N*	24	23	24									
	Median	0	0	0	4.23	2	.121	N/A					
	IQR	0	1	9.75									
RBQ—Insistence on Sameness	*N*	24	24	23									
	Median	0	0	0	1.39	2	.499	N/A					
	IQR	3	1	6									
TAQ—Impulsivity	*N*	24	24	23									
	Median	15.5	18	15	.552	2	.759	N/A					
	IQR	9.75	13.5	11									
TAQ—Overactivity	*N*	24	24	23									
	Median	18	19.5	19	.618	2	.734	N/A					
	IQR	8.75	14	13									
TAQ—Total	*N*	24	24	23									
	Median	33.75	35	32.5	.347	2	.841	N/A					
	IQR	15.25	22.9	25.8									

### Sensory experiences

The median scores of individuals with PTHS fell into the “deficient” range for *Hypo-Responsiveness* and *Social Contexts* and “at-risk” for *Hyper-Responsiveness, Sensory Seeking*, and *Non-Social Contexts* (see Table 8), according to Baranek’s [56] classifications based on normative data for typically developing children. The majority of individuals with PTHS were classified as “atypical” (defined as either “at risk” or “deficient” range), in relation to *Hypo-Responsiveness* (95.8%) and *Social Contexts* (91.7%).

**Table 8:** Number and percentage of individuals with PTHS scoring within the atypical range on the SEQ

*N* (%) within atypical range		PTHS, *N* = 24
Total SEQ	Median (IQR)	86.5 (15.98)
	*N* (%)	23 (95.80)
	Classification	At risk
Hypo-responsiveness	Median (IQR)	16.5 (6.25)
	*N* (%)	23 (95.80)
	Classification	Deficient
Hyper-responsiveness	Median (IQR)	31.2 (11.58)
	*N* (%)	15 (62.50)
	Classification	At risk
Sensory-seeking	Median (IQR)	42 (5.75)
	*N* (%)	18 (75)
	Classification	At risk
Social contexts	Median (IQR)	23.5 (7.25)
	*N* (%)	22 (91.70)
	Classification	Deficient
Non-social contexts	Median (IQR)	61.3 (11.50)
	*N* (%)	18 (75)
	Classification	At risk

Criterion cut-off points based on typically developing normative data [56]

Total: typical range (33–74), at risk range (75–86) and deficient range (87–165). Hypo-responsitivity: typical range (6–10), at risk range (11–12) and deficient range (13–30). Hyper-responsitivity: typical range (14–29), at risk range (30–34) and deficient range (35–70). Sensory seeking: typical range (13–38), at risk range (39–47) and deficient range (48–65). Social contexts: typical range (10–18), at risk range (19–21) and deficient range (22–50). Non-social contexts: typical range (22–55), at risk range (56–65) and deficient range (66–11)

### Challenging behaviour

A large proportion of the individuals with PTHS had displayed self-injurious behaviour (70.8%) and/or physical aggression (54.2%) in the last month; property destruction was also reported for 37.5% of the sample (see Table 9). A significantly greater proportion of individuals with PTHS showed self-injurious behaviour compared to individuals with AS (*χ*^2^ = 4.15, *p* = .042, RR = 1.7, 95% CI = .99–2.91), and a significantly greater proportion of individuals with PTHS showed physical aggression compared to individuals with CdLS (*χ*^2^ = 4.27, *p* = .039, RR = 2.17, 95% CI = .99–4.75). Fewer people with PTHS (37.5%) displayed destruction of property relative to AS (54.2%) or CdLS (54.2%), although these differences did not reach statistical significance.

**Table 9:** Number and percentage of individuals with PTHS and matched AS and CdLS displaying challenging behaviour and chi-squared analysis

	PTHS, *N* = 24	AS, *N* = 24	CdLS, *N* = 24	Chi-squared test			Post hoc comparison: chi-squared test				
				*χ* ^2^	df	*p* value	Comparison	*χ* ^2^	df	*p* value	Relative risk (95% CI)
Displayed SIB in the last month (%)	17 (70.8%)	10 (41.7%)	19 (79.2%)	8.07	2	.018	PTHS > AS	4.15	1	.042	1.70 (.99 to 2.91)^a^
Displayed destruction of property in the last month (%)	9 (37.5%)	13 (54.2%)	13 (54.2%)	1.78	2	.411					
Displayed physical aggression in the last month (%)	13 (54.2%)	16 (66.7%)	6 (25%)	8.79	2	.012	PTHS > CdLS	4.27	1	.039	2.17 (.99 to 4.75)^b^
Displayed stereotyped behaviour in the last month (%)	18 (75%)	16 (66.7%)	21 (87.5%)	2.93	2	.232					

^a^Relative risk of self-injurious behaviour in AS relative to PTHS

^b^Relative risk of physical aggression in CdLS relative to PTHS

### Mood

Individuals with PTHS displayed significantly lower scores than individuals with AS on the total MIPQ-S score (*U* = 185.5, *p* = .034, *r* = .31, medium effect size) and *Mood* subscale (*U* = 105, *p* < .001, *r* = .55, large effect size). No significant differences were evident between any of the groups on the *Interest and Pleasure* subscale (see Table 7).

### Activity

No significant differences were found between individuals with PTHS and individuals with AS or CdLS on the total TAQ score or *Impulsivity* or *Overactivity* subscales (see Table 7).

## Discussion

The current study used standardised informant report measures validated for people with ID and a cross-syndrome comparative approach, to further understand the behavioural profile in PTHS. To our knowledge, this is the first study using such methodology to explore the behavioural phenotype of PTHS.

The findings presented here are consistent with previous indications that a large majority of people with PTHS may meet criteria for ASD [14]. A very high proportion of individuals with PTHS in the current study (> 95%) met cut-off for ASD symptomatology on the SCQ. This significantly exceeded the proportion of individuals meeting cut-off in the group of matched individuals with AS, a syndrome associated with elevated likelihood of ASD [17]. Although a greater proportion of individuals with PTHS than CdLS met cut-off for ASD, this difference was not statistically significant. Notably, CdLS is a syndrome group with a well-established association with ASD [17], and therefore, this finding is consistent with high levels of ASD symptomatology in both groups. It is important to note that the SCQ is not a diagnostic tool and may over-estimate the prevalence of ASD in genetic syndrome groups, given that developmental level is not taken into account [20]. However, this is likely to be the case for all syndrome groups in the current analysis, given that their ability levels were approximately matched. Although the SCQ has a strong history in the elucidation of ASD-related behaviours in genetic syndrome groups associated with divergent ID profiles (e.g. [34, 67]), future studies should consider the use of additional measures to explore ASD phenomenology more comprehensively in PTHS.

Refining the ASD-related behavioural profile in PTHS based on the preliminary data we have presented here may have significant clinical implications regarding the utility of services for ASD in this syndrome group. ASD specific interventions may be useful for those diagnosed with co-occurring ASD, but the specific target of such interventions and their appropriateness in PTHS warrants further investigation. Future research may wish to explore the utility of ASD intervention models in this syndrome group (e.g. Applied Behaviour Analysis [68]) and whether existing ASD early intervention programs will also be of benefit in PTHS (e.g. JASPER, Joint Attention Symbolic Play Engagement and Regulation [69]).

It is important to consider the specific profile of ASD phenomenology in syndrome groups, as evidence suggests the profile of ASD-related behaviours may differ from idiopathic ASD (e.g. [70]) and may also vary considerably between syndrome groups (e.g. [16, 17]). In the case of fragile X syndrome, for instance, the pattern of repetitive behaviours (e.g. fewer compulsive and ritualistic behaviours) and social communication deficits (e.g. relatively intact social response, facial expression and social smile behaviours) appears to be qualitatively distinct from idiopathic ASD [71], alluding more to difficulties relating to social anxiety as opposed to social preference [72]. Behavioural comparisons to an idiopathic ASD group will further elucidate the profile of similarities and differences in PTHS.

The current data suggest difficulties with both social communication and restricted/repetitive behaviours in PTHS, supporting earlier literature presented by Zollino and colleagues [5]. Specifically, individuals with PTHS showed greater social communication deficits than those with AS and similarly high levels of impairment to those with CdLS (as outlined in the existing CdLS literature [19]). Further exploration of social characteristics using a dedicated sociability questionnaire, the SQID [31], confirmed lower levels of sociability in PTHS than in AS, but higher sociability with unfamiliar people in PTHS compared to CdLS. This may partially reflect high rates of social anxiety in CdLS [50, 51] that is more likely to manifest with unfamiliar people and potentially an absence of such social anxiety presentations in PTHS. However, the current study also found individuals with PTHS displayed lower mood than individuals with AS, partially contradicting the comparable happy demeanour and affectionate temperament in PTHS. Therefore, the association between internalising states and social characteristics warrants further investigation in PTHS.

Repetitive behaviours were comparable across all three syndrome groups in the current study. Most notably, rates of compulsive behaviour and insistence on sameness were markedly low in these groups, as previously reported in the AS and CdLS literature [61]. Specifically, a large proportion of the PTHS group (75%) showed object, body or hand stereotypies within the stereotyped behaviour domain, which are likely to contribute to the presentation of ASD-like characteristics in this group. Over 95% of the sample was reported to have displayed hypo-responsive behaviours within social contexts and over 60% displayed hyper-responsive behaviours to sensory input. Both hyper- and hypo-responsivity have previously been reported in children with ASD and developmental delay [35, 73], and therefore, this atypical presentation of sensory processing in individuals with PTHS may also relate to the ASD presentation in this syndrome. Given the established relationship between sensory processing deficits and stereotyped behaviours in the ASD literature [74], which may be mediated by anxiety in some cases [75], the distinct profile of stereotyped behaviours and its potential neuropsychological correlates should form a focus for future PTHS research.

High rates of physical aggression and self-injury in the PTHS group should also be explored at a functional level. Further research may aim to delineate potentially contributory factors to these behaviours, in relation to cognitive, biological and environmental correlates. It is possible, for example, that the sensory processing difficulties documented in this study may contribute to physical aggression (e.g. see [76]), and given the established relationship between self-injury and gastroesophageal reflux in CdLS [77], the potential contribution of pain to challenging behaviour in PTHS should not be underestimated. A majority of participants in the current cohort were reported to show gastrointestinal problems (83.3%), lending support for a more thorough and comprehensive behavioural assessment of pain in this syndrome group. Measures such as the Face, Legs, Activity, Cry and Consolability (FLACC) behavioural pain assessment scale [78] and the Non-Communicating Children’s Pain Checklist (NCCPC [79]) have been utilised effectively to explore the relationship between pain and behaviour in other syndrome groups (e.g. [80]) and may offer similar utility in PTHS.

This study employed a number of measures with known psychometric properties in ID research (e.g. [18, 19, 24, 31, 35]). However, the limitations associated with the use of informant-report behavioural questionnaires and a screening measure to explore ASD phenomenology should be held in mind. Future research would benefit from use of direct observational approaches and gold standard assessment tools, such as the Autism Diagnostic Observation Schedule (ADOS [81]) to explore specific topographies of behaviour in this syndrome group. More detailed measures of ability would also enable a more thorough matching strategy, to further delineate the contribution of ID, verbal ability and adaptive functioning to behaviour in PTHS.

Although the AS and CdLS groups are relatively representative of their syndromes described within literature (e.g. [12, 24, 25]), it should be noted that the matching process employed introduced selection bias for the comparison groups in this study, and thus, the AS and CdLS groups selected may not be wholly representative. The proportion of people meeting criteria for ASD in these groups exceeded that generally reported in the literature (43% CdLS, 34% AS) [17], which may be a consequence of individuals with lower levels of adaptive functioning being selected as matched participants for the PTHS group.

This study also did not employ direct genetic testing. All participants had a confirmed genetic mutation of the TCF4 gene as reported by parents/caregivers; however, it was not possible to delineate potential genotype-phenotype relationships. Results indicate possible behavioural differences between PTHS and AS groups, despite phenotypic similarities noted in the literature; this may be useful in distinguishing clinically between these syndromes and in appropriate targeting of diagnostic tests. Genetic analysis of both AS and PTHS can be complex, as a number of pathogenic variants involving the UBE3A gene and the TCF4 gene can lead to an AS or PTHS diagnosis, respectively [4, 39]. Routine sequencing or microarray analysis may not always be able to confirm diagnosis and further testing, for example testing for single exon deletions, might only be pursued if there is a strong clinical suspicion of either of these disorders, with behavioural features being one of the major distinguishing features. Given the deletion/non-deletion phenotypic distinctions in AS [82, 83], phenotypic differences relating to genetic subtype may be an important avenue for future PTHS research.

## Conclusion

Individuals with PTHS showed greater impairment in reciprocal social interaction and social communication than those with AS, with a greater proportion of those with PTHS than AS meeting cut-off scores for ASD symptomatology. Individuals with PTHS also evidenced high rates of stereotyped behaviour and atypical sensory processing, which may be further indicative of a profile of behaviour in PTHS which has features in common with ASD (see also [5, 14]). Future research should explore whether the profile of ASD-related characteristics is qualitatively convergent with that of individuals with idiopathic ASD via formal comparisons utilising direct behavioural observations and gold standard assessments.

## Data Availability

The data that support the findings of this study are not available due to them containing information that could compromise research participant consent.

## Abbreviations

ADHD: Attention deficit hyperactivity disorder
ADOS: Autism Diagnostic Observation Schedule
AS: Angelman syndrome
ASD: Autism spectrum disorder
CBQ: The Challenging Behaviour Questionnaire
CdLS: Cornelia de Lange syndrome
FLACC: Face, Legs, Activity, Cry and Consolability behavioural pain assessment scale
HQ: Health Questionnaire
ID: Intellectual disability
JASPER: Joint Attention Symbolic Play Engagement and Regulation
MIPQ: The Mood, Interest and Pleasure Questionnaire
NCCPC: Non-Communicating Children’s Pain Checklist
PTHS: Pitt-Hopkins syndrome
RBQ: The Repetitive Behaviour Questionnaire
SCQ: Social Communication Questionnaire
SEQ: Sensory Experience Questionnaire
SIB: Self-injurious behaviour
SQID: The Sociability Questionnaire for people with Intellectual Disability
TAQ: The Activity Questionnaire
WQ: Wessex Questionnaire
